# F9 Fimbriae of Uropathogenic *Escherichia coli* Are Expressed at Low Temperature and Recognise Galβ1-3GlcNAc-Containing Glycans

**DOI:** 10.1371/journal.pone.0093177

**Published:** 2014-03-26

**Authors:** Daniël J. Wurpel, Makrina Totsika, Luke P. Allsopp, Lauren E. Hartley-Tassell, Christopher J. Day, Kate M. Peters, Sohinee Sarkar, Glen C. Ulett, Ji Yang, Joe Tiralongo, Richard A. Strugnell, Michael P. Jennings, Mark A. Schembri

**Affiliations:** 1 Australian Infectious Diseases Research Centre, School of Chemistry and Molecular Biosciences, The University of Queensland, Brisbane, Australia; 2 Institute for Glycomics, Griffith University, Gold Coast, Queensland, Australia; 3 School of Medical Sciences, Centre for Medicine and Oral Health, Griffith University, Southport, Queensland, Australia; 4 Department of Microbiology and Immunology, The University of Melbourne, Parkville, Victoria, Australia; Centre National de la Recherche Scientifique, Aix-Marseille Université, France

## Abstract

Uropathogenic *Escherichia coli* (UPEC) is the leading causative agent of urinary tract infections (UTI) in the developed world. Among the major virulence factors of UPEC, surface expressed adhesins mediate attachment and tissue tropism. UPEC strains typically possess a range of adhesins, with type 1 fimbriae and P fimbriae of the chaperone-usher class the best characterised. We previously identified and characterised F9 as a new chaperone-usher fimbrial type that mediates biofilm formation. However, the regulation and specific role of F9 fimbriae remained to be determined in the context of wild-type clinical UPEC strains. In this study we have assessed the distribution and genetic context of the *f9* operon among diverse *E. coli* lineages and pathotypes and demonstrated that *f9* genes are significantly more conserved in a UPEC strain collection in comparison to the well-defined *E. coli* reference (ECOR) collection. In the prototypic UPEC strain CFT073, the global regulator protein H-NS was identified as a transcriptional repressor of *f9* gene expression at 37°C through its ability to bind directly to the *f9* promoter region. F9 fimbriae expression was demonstrated at 20°C, representing the first evidence of functional F9 fimbriae expression by wild-type *E. coli*. Finally, glycan array analysis demonstrated that F9 fimbriae recognise and bind to terminal Galβ1-3GlcNAc structures.

## Introduction

Urinary tract infections (UTI) are among the most common infectious diseases of humans and a major cause of morbidity. In the USA, UTI account for approximately $1.6 billion in medical expenditures each year [1]. It is estimated that 40–50% of adult healthy women will experience at least one UTI episode in their lifetime. The recurrence rate of UTI is high and often the infections tend to become chronic with many subsequent episodes. UTIs usually start as cystitis but often evolve to encompass the kidneys and can ultimately result in dissemination into the bloodstream and/or renal failure. Catheter-associated UTIs are also very common and account for 40% of all nosocomial infections. Most patients with an indwelling urinary catheter for thirty days or more develop bacteriuria [2].

Uropathogenic *Escherichia coli* (UPEC) is the cause of the majority (>80%) of UTIs in humans. UPEC isolates contain numerous virulence factors, which allow for the successful colonisation of the urinary tract. Although no single virulence factor is uniquely definitive of UPEC, the ability to cause symptomatic UTI is enhanced by adhesins (e.g. type 1 and P fimbriae) and toxins (e.g. hemolysin) [3], [4]. Adherence to the urinary tract epithelium is the first stage of UTI as it enables bacteria to resist the hydrodynamic forces of urine flow and establish infection. Among the best-described adhesins produced by UPEC are type 1, P, and F1C/S fimbriae of the chaperone-usher (CU) pathway [4].

The CU pathway is a highly conserved secretion system in Gram-negative bacteria that mediates the assembly of hair-like fimbrial polymers on the bacterial cell surface. CU fimbrial biogenesis requires a dedicated periplasmic chaperone and an outer membrane usher protein that functions as an assembly platform of the fimbrial organelle which is primarily composed of a helical array of 500 to 3,000 copies of major subunit protein [5], [6]. The receptor-binding adhesin resides at the distal end of the fimbrial organelle and contains a C-terminal domain which connects the adhesin to the terminal major subunit protein sometimes aided by one or more minor subunits, and an N-terminal lectin domain which mediates binding to specific ligands [3]. The genes encoding the various components of CU fimbriae are typically organised in an operon and transcribed as a single polycistronic mRNA molecule [7].

Genomic analysis of the *E. coli* pan genome has revealed 38 distinct chaperone-usher fimbrial types based on genomic locus position and usher phylogeny [8]. Type 1 and P fimbriae are primary contributors to the colonisation of the urinary tract by UPEC and have been the focus of extensive study (for a review, refer to [9]). Type 1 fimbriae confer binding to α-D-mannosylated proteins such as uroplakins, which are abundant in the uroepithelial lining of the bladder [10]. P fimbriae contribute to UTI by binding to the α-Gal(1–4)β-Gal receptor epitope in the globoseries of glycolipids found in the kidney [11], [12]. F1C/S fimbriae also contribute to UTI through their ability to bind to GalNAcβ1-4Galβ glycolipids and sialyl galactoside glycoproteins present on epithelial cells in the bladder and kidneys [13]–[15].

We previously characterised F9 fimbriae as a new CU fimbriae type in UPEC [16]. F9 fimbriae are part of the γ1 fimbrial subclade and are closely related to type 1 and F1C/S fimbriae in genetic organization and structural composition [8], [17]. Low levels of expression of the F9 major subunit have been detected in enterohemorrhagic *E. coli* (EHEC) strain O157:H7 EDL933 and in a UPEC CFT073*fim foc* null-mutant, however, to date there is no evidence of functional F9 fimbriae expression in any wild-type *E. coli* strain [16], [18]. Cloning and expression of the *f9* genes in a recombinant *E. coli* strain revealed F9 fimbriae mediate strong biofilm formation, however F9 expression did not confer hemagglutination or cellular adherence properties. In this study, we have examined the distribution and conservation of the *f9* operon in *E. coli*, demonstrated the high frequency of *f9* genes in extant *E. coli* strains, and evaluated the conservation of the F9 adhesin lectin domain. Additionally, we have demonstrated that the *f9* fimbrial gene cluster is subjected to temperature-dependent repression by the global regulator H-NS. Repression was alleviated at lower temperatures, at which F9 fimbriae mediated significant biofilm formation on abiotic surfaces by wild-type *E. coli.* In order to study the ligand recognition properties of F9 fimbriae, we utilized a glycan array and identified Galβ1-3GlcNAc and lacto-*N*-tetraose glycans as novel receptor targets for these fimbriae.

## Materials and Methods

### Phylogenetic inference, sequence alignment and diversity estimations

The complete genomes of 42 *E. coli* strains representing the diversity of the species were investigated for presence of the *f9* operon using the NCBI BLAST2.2.25+ program [19]. The *f9* operon and its genomic context were aligned and visualised using Easyfig [20]. The evolutionary relationship of the 42 *E. coli* strains was predicted by Multi-Locus Sequence Typing (MLST) of the ∼9 kb concatenated nucleotide sequences of 7 housekeeping genes (*adk*, *fumC*, *gyrB*, *icd*, *mdh*, *purA*, *recA*) as previously described [21]. Sequences were aligned in MEGA5 using the ClustalW DNA weight matrix under default settings. The Neighbour-Joining method of MEGA5 was used to infer the evolutionary history, with distances computed by the Jukes-Cantor method. The resulting phylogenetic tree was tested by a bootstrap test of 1000 replicates and visualised in iTOL [22] as a rooted phylogram. Diversity of the F9 adhesin protein was estimated with MEGA5. The mature adhesin (280 aa) and adhesin lectin domain (160 aa) sequences were aligned with ClustalW using the BLOSUM protein weight matrix under default settings; diversity was calculated using the Poisson model with a bootstrap confidence test for standard error measurements (1000 replicates).

### Bacterial strains, plasmids and culture conditions

Strains and plasmids used in this study are listed in Table 1. *E. coli* CFT073 was isolated from the blood and urine from a woman with acute pyelonephritis [23]. *E. coli* strains were routinely cultured at 37°C on solid or in liquid lysogeny broth (LB) medium [24] or liquid M9 minimal medium (42 mM Na_2_HPO_4_, 22 mM KH_2_PO_4_, 9 mM NaCl, 18 mM NH_4_Cl, 1 mM MgSO_4_, 0.1 mM CaCl_2_ and 0.2% (w/v) glucose). Where appropriate, media were supplemented with ampicillin (100 μg ml^−1^), kanamycin (100 μg ml^−1^) or chloramphenicol (25 μg ml^−1^). To induce expression of F9 fimbriae from plasmid pF9, culture media were supplemented with 0.2% (w/v) arabinose. Plasmid transformations into *E. coli* CFT073 were mediated by electroporation.

**Table 1:** Strains and plasmids used in this study.

*E.coli* Strain or Plasmid	Relevant Characteristics	Reference
**Strain**		
CFT073	Wild-type UPEC reference strain	Welch *et al*. 2002 [52]
CFT073*f9*	CFT073 *c1931-c1936*::*kan*, Kan^r^	Ulett *et al.* 2007 [16]
CFT073*hns*	CFT073 *c1701*::*kan*, Kan^r^	Allsopp *et al.* 2012 [33]
CFT073*f9 hns*	CFT073 *c1931-c1936*, *c1701*::*kan*, Kan^r^	This study
CFT073*Δ4*	CFT073*fim foc pap1 pap2*	This study
CFT073*Δ4 f9*	CFT073*fim foc pap1 pap2, c1931-c1936*::*kan*, Kan^r^	This study
CFT073*Δ4 hns*	CFT073*fim foc pap1 pap2, c1701*::*kan*, Kan^r^	This study
CFT073*Δ4 f9 hns*	CFT073*fim foc pap1 pap2, c1931-c1936, c1701*::*kan*, Kan^r^	This study
CFT073*virF*-like	CFT073 *c0421*::*kan*, Kan^r^	Allsopp *et al.* 2012 [33]
CFT073*rpoS*	CFT073 *c1699*::*kan*, Kan^r^	Allsopp *et al.* 2012 [33]
CFT073*virF*-like	CFT073 *c2091*::*kan*, Kan^r^	Allsopp *et al.* 2012 [33]
CFT073*hns*-like	CFT073 *c2411*::*kan*, Kan^r^	Allsopp *et al.* 2012 [33]
CFT073*stpA*	CFT073 *c3218*::*kan*, Kan^r^	Allsopp *et al.* 2012 [33]
CFT073*luxS*	CFT073 *c3244*::*kan*, Kan^r^	Allsopp *et al.* 2012 [33]
CFT073*virF*-like	CFT073 *c3744*::*kan*, Kan^r^	Allsopp *et al.* 2012 [33]
CFT073*cpxR*	CFT073 *c4864*::*kan*, Kan^r^	Allsopp *et al.* 2012 [33]
CFT073*soxR*	CFT073 *c5054*::*kan*, Kan^r^	Allsopp *et al.* 2012 [33]
MS428	K-12 MG1655*fim*	Kjaergaard *et al.* 2000 [53]
**Plasmids**		
pKD4	Template for *kan* gene amplification, Kan^r^	Datsenko & Wanner 2000 [26]
pKD46	λ-Red recombinase expression vector, Amp^r^	Datsenko & Wanner 2000 [26]
pCP20	Temperature sensitive FLP expression vector, Amp^r^	Datsenko & Wanner 2000 [26]
pBAD30	Cloning vector with *ara* promoter, Amp^r^	Guzman *et al.* 1995 [54]
pF9	c1931-c1936 (F9^CFT073^)in pBAD30, Amp^r^	Ulett *et al.* 2007 [16]
pBR322	Cloning vector, Amp^r^, Tet^r^	Bolivar *et al.* 1977 [55]
pH-NS	c1701 (H-NS^CFT073^) in pBR322, Amp^r^	Allsopp *et al.* 2012 [33]
pACYC184	Cloning vector, Cam^r^, Tet^r^	Chang & Cohen 1978 [56]
pDW11	*gfp* (GFP^pKEN2^) in pACYC184, Cam^r^	This study

### DNA manipulations and genetic techniques

Plasmid DNA was isolated using the QIAprep Spin Miniprep kit (Qiagen). Chromosomal DNA was purified using the GenomicPrep cell and tissue DNA isolation kit (GE Healthcare Life Sciences). PCR was performed using *Taq* DNA polymerase according to manufacturer's instructions (Roche). Restriction endonucleases were used according to the manufacturer's specifications (New England Biolabs). Oligonucleotide primers used in this study were purchased from Sigma-Aldrich and are listed in Table S1. For sequencing, PCR products were amplified using the BigDye Terminator v3.1 Cycle DNA Sequencing Kit according to manufacturer's specifications (AB SCIEX), and analysed subsequently by the Australian Equine Genome Research Centre.

### Construction of plasmid pDW11

To generate a Green Fluorescent Protein (GFP) expressing plasmid compatible with pF9, the *gfp* gene from plasmid pKEN2 [25] was amplified with primers 2319 and 2320 containing 5′ BamHI and SalI sites, respectively (Table S1). The PCR product was digested by BamHI and SalI and directionally cloned into the corresponding sites of cloning vector pACYC184. Plasmid transformed *E. coli* strains were screened for GFP expression by fluorescence microscopy.

### Construction of CFT073 gene deletion mutants

*E. coli* CFT073 gene deletion mutants were constructed using the λ-Red mediated homologous recombination system as previously described [26]. Briefly, the FRT-flanked kanamycin resistance gene from pKD4 was amplified using primers containing 5′ 50 bp regions homologous to the start and end sequence of the gene(s) to be deleted. The resulting approximately 1.6 kb PCR products were introduced by electroporation into appropriate strains expressing λ-Red recombinase from pKD46. Kanamycin resistant colonies were analysed by PCR and DNA sequencing to confirm deletion of the relevant gene. For the construction of *E. coli* CFT073 isogenic null-mutants, the kanamycin gene was removed using the pCP20 FLP-FRT site-specific recombination system, allowing for successive rounds of mutagenesis [26].

### Protein immunoblotting

Rabbit immune serum against an F9 fimbriae over-expressing *E. coli* strain was previously generated [16]. Sera were absorbed against cell lysates of *f9* negative stains and *f9* null-mutants. For western blot analysis, bacterial EDTA heat-induced outer membrane vesicles (OMVs) were generated to enrich for the outer membrane-associated protein fraction, using a previously described method [27] with several modifications. Briefly, 50 ml LB or M9 medium was inoculated with 100 μl pre-culture (grown in the same medium) and incubated for 18 h at 37°C, 28°C or 20°C 250 rpm. Cells were harvested at 10,000×*g* for 10 min at 4°C and washed in 25 ml 4°C PBS. The bacterial pellet was resuspended in 1 ml EDTA buffer (0.05 M Na_2_HPO_4_, 0.15 M NaCl, 0.01 M ethylenediaminetetraacetic acid (EDTA), pH 7.4) and incubated 30 min at 56°C, statically. Cells were centrifuged at 10,000×*g* for 10 min at 4°C and the supernatant was filtered using a 0.22 μm PVDF low protein binding filter (Millipore). Trichloroacetic acid (TCA) was added to a final concentration of 20% (w/v) to precipitate proteins overnight at 4°C. Protein suspensions were separated according to electrophoretic mobility using SDS-PAGE and transferred to a polyvinylidene difluoride (PVDF) membrane, which was subsequently incubated in 1∶500 rabbit polyclonal absorbed anti-F9 primary sera, followed by 1∶10,000 goat anti-rabbit immunoglobulin G-alkaline phosphatase-conjugated secondary antibody (Sigma-Aldrich). Signal development was performed using the substrate 5-bromo-4-chloro-3-indolylphosphate–nitroblue tetrazolium (BCIP/NBT; Sigma-Aldrich).

### 5′ Rapid amplification of cDNA ends (5′ RACE)

The transcription start site of the *f9* operon was determined using the 5′ RACE System v2.0 (Invitrogen) [28]. Experiments were performed according to manufacturer's specifications except for the modifications listed below. Three gene specific primers were used for this assay: 4235, 4236 and 4237 (Table S1). To verify that the first nucleotide of the mRNA was a guanine, the cDNA was dA-tailed with a dATP substitution. PCR amplification of dA-tailed cDNA was performed using the (dT)17-adaptor primer 4296 (Table S1). Amplified cDNA was sequenced by the Australian Equine Genome Research Centre.

### Electrophoretic mobility shift assay

Gel shift assays were performed as previously described [29]. Briefly, a DNA mixture containing an equimolar ratio of the 251 bp PCR amplified *f9* promoter region and *Taq*I-*Ssp*I digested pBR322 was incubated with native purified H-NS protein in 30 μl H-NS binding buffer (40 mM HEPES pH 8, 60 mM potassium glutamate, 8 mM magnesium aspartate, 5 mM dithiothreitol, 10% glycerol, 0.1% octylphenoxypolyethoxyethanol, 0.1 mg/ml bovine serum albumin) for 15 min at room temperature. DNA fragments and DNA-protein complexes were resolved by gel electrophoresis (0.5× Tris/Borate/EDTA buffer, 3% agarose MS gel, ran at 50 V, 4°C), stained with ethidium bromide and visualised by ultraviolet illumination.

### Microtitre plate biofilm formation assay

Bacterial biofilm formation was assessed on sterile non-coated 96-well polyvinyl chloride (PVC) microtitre plates (BD Falcon) as previously described [30]. Briefly, cells were cultured with aeration at various temperatures for 24 hours in 150 μl M9 medium containing 0.2% (w/v) glucose. After incubation, cells were washed, stained with 0.1% crystal violet for 30 min at 4°C, and washed three additional times. Bound bacterial cells were quantified by adding ethanol-acetone (80∶20 v/v) and measurement of the dissolved crystal violet at an optical density of 595 nm.

### F9 Immunogold labelling and electron microscopy

Cells for immunogold labelling and transmission electron microscopy (TEM) were prepared from liquid cultures grown overnight at 20°C in M9 minimal medium. A glow-discharged carbon-coated Formvar copper grid was placed on a drop of the bacterial suspension for 1 min to allow the cells to adsorb. Grids were washed twice on drops of water (1 min), and blocked for 30 min in blocking buffer (PBS containing 0.2% BSA, 0.2% fish skin gelatin, 20 mM glycine). Samples were exposed to 1∶25 anti-F9 rabbit immune serum for 30 min and washed four times in blocking buffer (5 min) before incubation with Protein A-gold conjugate (10 nm diameter, diluted 1∶60 in blocking buffer) for 30 min and four washes (5 min) in PBS. Cells were fixed with 4% paraformaldehyde in PBS (5 min) and grids were washed four times (2 min) in sterile ultrapure water before examination under a JEOL 1010 TEM operated at 80 kV. Images were captured using an analySIS Megaview III digital camera.

### Glycan array analysis

Glycan array slides and whole-cell binding assays were essentially performed as previously described [31], [32]. Glycan arrays comprised 120 unique carbohydrates (Table S2) printed on super epoxy slides (Arrayit). All array experiments consisted of a minimum of three independent biological repeats. *E. coli* strains MS428 (pF9, pDW11) and MS428 (pBAD, pDW11) were cultured overnight at 37°C with gentle agitation in M9 minimal medium containing ampicillin (100 μg ml^−1^), chloramphenicol (25 μg ml^−1^) and 0.2% arabinose. Post-incubation, cells were diluted to an OD of 0.6, representing approximately 1×10^7^ CFU ml^−1^. A volume of 125 μL of cells was hybridised to a pre-blocked (0.1% BSA in PBS with 2 mM MgCl_2_ and CaCl_2_, 5 mins) glycan array for 20–30 mins at room temperature in the dark. Glycan array slides were placed in a 50 mL tube and washed in filter sterilized buffer 1 (PBS with 2 mM MgCl_2_ and CaCl_2_) for 5 mins, buffer 2 (buffer 1 with 0.01% tween-20) for 2 mins, and finally rinsed in fresh buffer 1. Cells were fixed for 10 mins in PBS with 10% formaldehyde and dried by centrifugation for 5 mins at 200×*g*. The array slide was scanned using a ProScan Array microarray scanner (Perkin Elmer) using a 488 nm argon laser. Images were attained and analysed using the ScanArray Express software package (Perkin Elmer). To determine minimum binding concentration, glycans were printed in serial dilutions from 5 mM to 5 fM on an array slide. For competition assays, cells were pre-treated with the glycan of interest for 15 mins prior to hybridisation on the slide.

### Statistical analyses

The frequency of intact *f9* operons in different *E. coli* strain collections and between different *E. coli* phylogenetic groups was compared using Fisher's exact test with a two-tailed *P* value. Biofilm formation was compared between *f9* encoding strains and their isogenic *f9* null mutant using a two-tailed *t* test. *P* values <0.05 were considered significant. For glycan array analysis, binding was classified as RFU (relative fluorescence units) above average background (defined as background mean plus 3 standard deviations) and was tested for statistical significance using a two-tailed *t* test with a *P* value <0.001.

## Results

### Genetic organisation and distribution of *f9* fimbrial operons in *E. coli*

**Figure 1:**
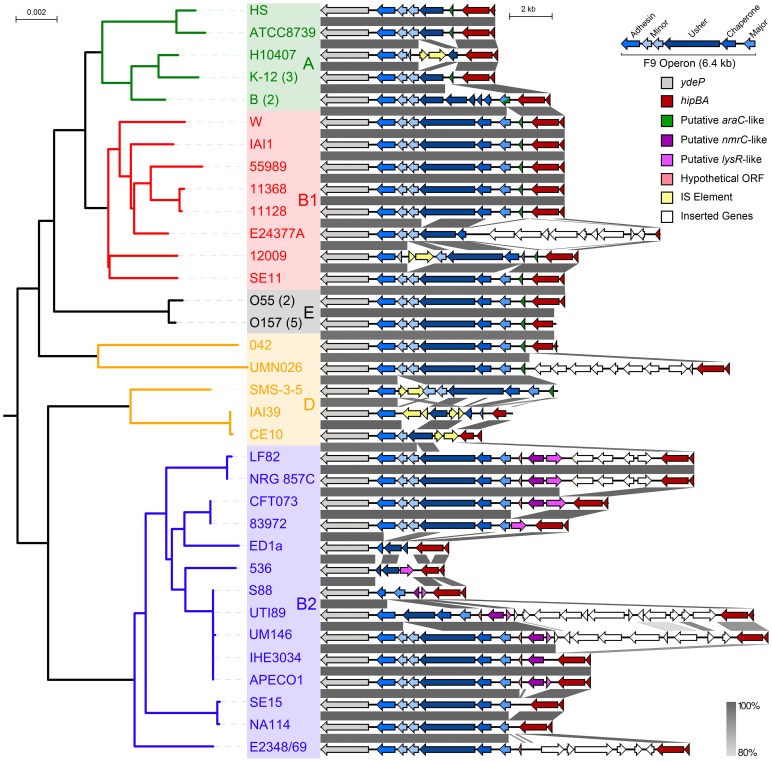
Conservation and genetic organisation of the *E. coli f9* fimbrial operon in an evolutionary context. Left: The phylogeny of 42 *E. coli* strains is displayed as inferred by the Neighbour-Joining method on the concatenated nucleotide sequence of 7 housekeeping genes (9,015 nt over an equal number of positions). *E. coli* strains are colour-coded according to phylogroup (A, B1, B2, D and E). The scale on the phylogenetic tree represents the number of nucleotide substitutions per site. Closely related strains with identical *f9* genetic context are collapsed and included *E. coli* K-12 (*n* = 3; strains MG1655, DH10β, BW2952), *E. coli* B (*n* = 2; strains BL21(DE3), B REL606), *E. coli* O55 (*n* = 2; strains CB9615, RM12579), *E. coli* O157 (*n* = 5; strains EDL933, Sakai, EC4115, TW14359, Xuzhou21). Right: Alignment of the *f9* genes (blue) and their flanking genes. The *f9* operon is flanked 3′ by the highly conserved *ydeP* gene (grey) and 5′ by the *hipBA* operon (red). The direct 5′ region of the *f9* operon is variable, and involves three distinct hypothetical transcriptional regulators (green, purple, and lilac). The percentage DNA sequence identity is indicated in grey. The scale on the aligned genetic context represents DNA length in kilobase pair.

In order to investigate the distribution and genetic location of the *f9* operon in *E. coli*, we examined the genome sequence of 42 diverse *E. coli* strains available on the NCBI database, including representatives of all *E. coli* lineages and various pathotypes (Table 2). The *E. coli* species exhibits extensive genetic substructure and can be divided into 5 major monophyletic clades (phylogroup A, B1, B2, D and E) [21]. To evaluate the conservation and evolutionary history of F9 fimbriae among *E. coli* phylogroups, a phylogenetic tree based on multi-locus sequence typing (MLST) of 7 concatenated housekeeping genes (∼9 kb) was constructed and combined with *f9* genomic context alignments (Figure 1). The *f9* operon consists of six structural genes, encoding, from 5′ to 3′: the major subunit, chaperone, usher, two minor subunits and an adhesin. *f9* operons containing deletions, truncations and/or insertion elements were considered disrupted. Comparative genomic analysis revealed that all strains possessed at least part of the F9 encoding DNA sequences. In 60% (25/42) of *E. coli* strains the *f9* operon appeared intact (Figure 1 and Table 2). Among *E. coli* phylogenetic groups, the *f9* operon was conserved in the majority of B1 and E strains, and to a lesser extent in B2 and D strains. The *f9* operon was disrupted in all phylogroup A strains. In a pathotype context, the intact *f9* operon was highly prevalent in intestinal pathogenic *E. coli*, including adherent-invasive *E. coli* (AIEC; 3/3), enteroaggregative *E. coli* (EAEC; 2/2), enteropathogenic *E. coli* (EPEC; 3/3) and enterohemorrhagic *E. coli* (EHEC; 7/8), but not in enterotoxigenic *E. coli* (ETEC; 0/2) (Table 2). The *f9* encoding genes were not detected in genome sequences from other bacterial genera available in the NCBI database (except for *Shigella*, a subgenus of *Escherichia*).

**Table 2:** *E. coli* genomes analysed in this study.

E.coli Strain	Phylogroup	F9 Status*	Reference
**UPEC**			
CFT073	B2	+	Welch et al. 2002 [52]
NA114	B2	+	Avasthi et al. 2011 [57]
UTI89	B2	D	Chen et al. 2006 [58]
536	B2	D	Hochhut et al. 2006 [59]
IAI39	D	D	Touchon et al. 2009 [60]
UMN026	D	+	Touchon et al. 2009 [60]
**ABU**			
83972	B2	+	Zdziarski et al. 2010 [61]
**NMEC**			
IHE3034	B2	+	Moriel et al. 2009 [62]
S88	B2	D	Touchon et al. 2009 [60]
CE10	D	D	Lu et al. 2011 [63]
**APEC**			
APEC01	B2	+	Johnson et al. 2007 [64]
**AIEC**			
LF82	B2	+	Miquel et al. 2010 [65]
NRG 857C	B2	+	Nash et al. 2010 [66]
UM146	B2	+	Krause et al. 2011 [67]
**EAEC**			
55989	B1	+	Touchon et al. 2009 [60]
042	D	+	Chaudhuri et al. 2010 [68]
**EPEC**			
O127:H6 E2348/69	B2	+	Iguchi et al. 2009 [69]
O55:H7 CB9615	E	+	Zhou et al. 2010 [70]
O55:H7 RM12579	E	+	Kyle et al. 2012 [71]
**ETEC**			
O78:H11 H10407	A	D	Crossman et al. 2010 [72]
E24377A	B1	D	Rasko et al. 2008 [73]
**EHEC**			
O26:H11 11368	B1	+	Ogura et al. 2009 [74]
O103:H2 12009	B1	D	Ogura et al. 2009 [74]
O111:H- 11128	B1	+	Ogura et al. 2009 [74]
O157:H7 EDL933	E	+	Perna et al. 2001 [75]
O157:H7 Sakai	E	+	Hayashi et al. 2001 [76]
O157:H7 EC4115	E	+	Eppinger et al. 2011 [77]
O157:H7 TW14359	E	+	Kulasekara et al. 2009 [78]
O157:H7 Xuzhou21	E	+	Xiong et al. 2012 [79]
**Environmental**			
SMS-3-5	D	D	Fricke et al. 2008 [80]
**Commensal**			
ATCC 8739	A	D	Joint Genome Institute [81]
HS	A	D	Rasko et al. 2008 [73]
IAI1	B1	+	Touchon et al. 2009 [60]
SE11	B1	+	Oshima et al. 2008 [82]
W	B1	+	Archer et al. 2011 [83]
ED1a	B2	D	Touchon et al. 2009 [60]
SE15	B2	+	Toh et al. 2010 [84]
**Laboratory**			
BL21(DE3)	A	D	Jeong et al. 2009 [85]
B REL606	A	D	Jeong et al. 2009 [83]
K-12 MG1655	A	D	Blattner et al. 1997 [86]
K-12 DH10β	A	D	Durfee et al. 2008 [87]
K-12 BW2952	A	D	Ferenci et al. 2009 [88]

UPEC: uropathogenic *E. coli*, ABU: asymptomatic bacteriuria *E. coli*, NMEC: neonatal meningitis *E. coli*, APEC: avian pathogenic *E. coli*, AIEC: adherent-invasive *E. coli*, EAEC: enteroaggregative *E. coli*, EPEC: enteropathogenic *E. coli*, ETEC: enterotoxigenic *E. coli*, EHEC: enterohaemorrhagic *E. coli*. *F9 status: + intact operon, D disrupted operon.

The *f9* operon is flanked by the highly conserved *ydeP* gene involved in acid resistance (downstream) and the relatively well-conserved *hipBA* cell persistence gene cluster (upstream). The immediate 5′ region is variable, and contains a range of different insertions and/or deletions, including three hypothetical transcriptional regulators (Figure 1). Currently, there is no evidence linking these putative regulators to the transcriptional control of *f9* genes.

### Prevalence of *f9* genes in *E. coli*

**Figure 2:**
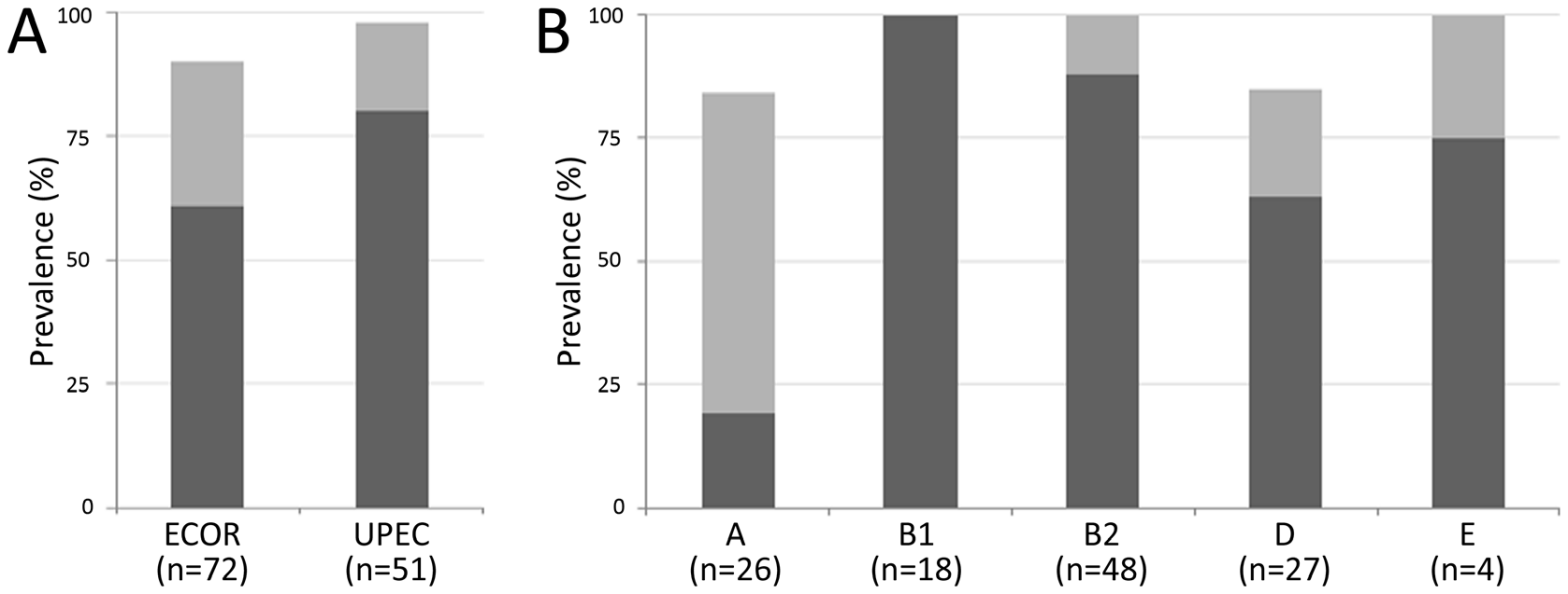
Prevalence of *f9* genes in *E. coli*. Strains of the *E. coli* reference ECOR (*n* = 72) and urosepsis UPEC (*n* = 51) collections were screened by PCR for *f9* major subunit, usher and adhesin genes. Bars in dark grey represent strains screening positive for all genes screened for (indicating the presence of an intact *f*9 operon), light grey bars indicate presence of at least one of the screened genes. *f9* genes are pervasive in *E. coli*, albeit not exclusively in an intact polycistronic conformation. (A) Intact *f9* operons are signifcantly more prevalent in UPEC strains (80%) than ECOR strains (61%)(*P*<0.05). (B) To evaluate F9 prevalence in a evolutionary context, strains from both collections were categorised according to phylogenetic group. Intact *f9* operons were prevalent in 100% of phylogroup B1 strains, and moderatly prevalent in strains belonging to phylogroups B2, D and E. The frequency of an intact *f9* operon was signifanctly lower in phylogenetic group A in comparison to the other phylogroups (*P*<0.05).

Based on the genomic analysis described above, primers were designed in conserved regions of the *f9* gene cluster to screen for the major subunit, usher and adhesin genes in two large *E. coli* strain collections. These included fifty-one UPEC isolates collected from patients presenting with urosepsis at the Princess Alexandra Hospital (Brisbane, Australia) as well as seventy-two strains of the well-defined and diverse ECOR reference collection. In the UPEC collection, 80% (41/51) of strains screened positive by PCR for all three *f9* genes (Figure 2A). A further 16% (8/51) screened positive for at least one *f9* gene, while 4% (2/51) of strains screened negative for all genes. In the ECOR collection, 61% (44/72) of strains screened positive for all three *f9* genes, 29% (21/72) screened positive for at least one *f9* gene and 11% (8/72) did not yield any positive PCR results. F9 operon prevalence (as judged by screening positive for the major subunit, usher and adhesin genes) was significantly higher (*P*<0.05) in UPEC isolates compared to the ECOR collection (Figure 2A). PCR data from the two *E. coli* collections were merged to evaluate *f9* prevalence relative to strain phylogenetic group (Figure 2B). Consistent with the genome-sequenced strains, the frequency of intact *f9* operons in phylogroup A strains was significantly lower in comparison to strains belonging to other phylogroups (*P*<0.05). The F9 operon was detected in 100% of phylogroup B1 strains and the majority (>70%) of strains from phylogroup B2, D or E.

### Transcription of the *f9* operon is repressed by H-NS

**Figure 3:**
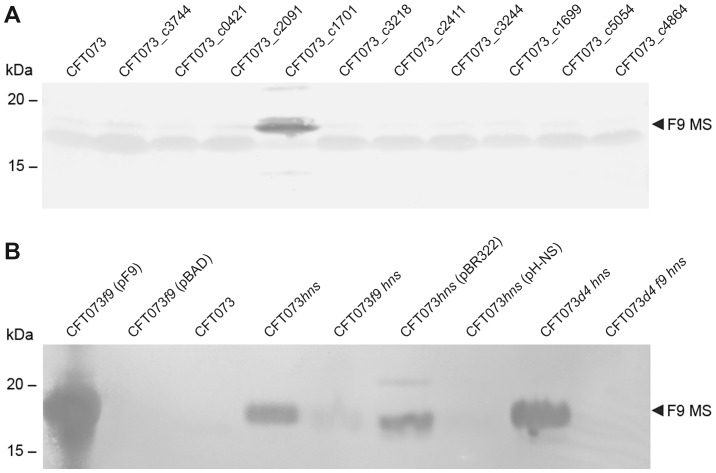
H-NS is a negative regulator of F9 fimbriae expression. (A) Western blot analysis of CFT073 and 10 isogenic defined/putative regulatory gene deletion mutants using an F9 specific antiserum. A strong-reacting band consistent with the size of the mature F9 major subunit (∼18.3 kDa, indicated by an arrow) was observed in CFT073 *hns* (CFT073_c1701) but not in the other regulator deletion mutants. (B) Western blot analysis of F9 fimbriae expression in CFT073 *f9* and *hns* null mutants cultured at 37°C. The F9 specific antiserum reacted strongly with the mature F9 major subunit (F9 MS indicated by an arrow, ∼18.3 kDa) in over-expressing strain CFT073*f9* (pF9). Repression of the *f9* operon is alleviated in the CFT073*hns* mutant. This signal is lost again in isogenic null mutant CFT073*f9 hns* or in the H-NS over-expressing complemented strain CFT073*hns* (pH-NS), demonstrating F9 fimbriae expression is negatively regulated by H-NS. The faint band in CFT073*f9 hns* suggests cross reactivity with a related fimbrial subunit, and is indeed lost in the isogenic *fim, foc, pap1, pap2* null mutant CFT073*Δ4f9 hns*.

We previously detected very weak expression of F9 fimbriae in UPEC strain CFT073*fim foc* cultured at 37°C [16]. However, to date there have been no reports of functional F9 fimbriae expression in wild-type UPEC. To investigate the genetic basis of *f9* gene regulation in UPEC, we employed a previously described panel of CFT073 isogenic single gene deletion mutants lacking a selection of defined/putative regulatory genes (c0421 [*virF*_like], c1699 [*rpoS*], c1701 [*hns*], c2091 [*virF*_like], c2411 [*hns*_like], c3218 [*stpA*], c3244 [*luxS* AI-2], c3744 [*virF*_like], c4864 [*cpxR*] and c5054 [*soxR*]) [33]. Expression of the F9 fimbrial major subunit protein was assessed by western blot analysis of CFT073 wild-type and mutant strains employing an F9 specific antiserum. In this experiment, strong expression of the F9 major subunit protein was only detected in the CFT073*hns* mutant strain, but not in any of the other regulator mutants following growth in LB broth at 37°C (Fig. 3A). To confirm these results, the *hns* mutant strain (referred to as CFT073*hns*) was complemented with the H-NS expressing plasmid pH-NS. No detectable F9 major subunit protein was detected in CFT073*hns*(pH-NS) (Figure 3B). Additionally, the strong F9 major subunit signal was absent in a CFT073*f9 hns* double mutant. Interestingly, a faint band similar in size to the F9 major subunit was observed in CFT073*f9 hns*, suggesting some non-specific cross reactivity of the F9 antiserum with a similar sized protein. Since H-NS negatively controls the expression of various distinct fimbrial operons, this observation could be the result of alleviation of repression of an F9 related fimbrial type [34]. We addressed this by constructing a mutant deleted for gene clusters encoding type 1, F1C, P1 and P2 fimbriae (referred to as CFT073*Δ4*), and a CFT073*Δ4* strain deleted for the *f9* genes (CFT073*Δ4 f9*). Indeed, mutation of the *hns* gene in CFT073*Δ4* and CFT073*Δ4 f9* resulted in the complete loss of this cross-reacting band (Figure 3B). Combined, these results demonstrate that H-NS represses the expression of F9 fimbriae in CFT073.

### H-NS binds to the promoter region of the *f9* operon

**Figure 4:**
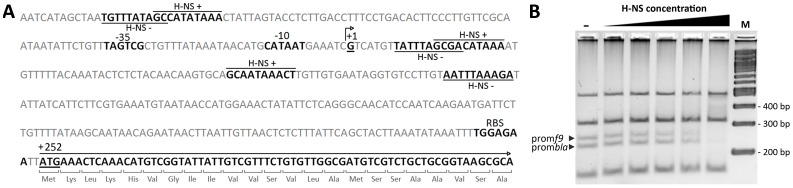
The H-NS protein binds to the *f9* promoter region. (A) Nucleotide sequence and features of the F9 promoter region of uropathogenic *E. coli* CFT073. 5′ RACE analysis identified the transcription start site as a guanine residue (labelled as +1), 251 nucleotides upstream of the start codon of the *f9* major subunit (+252). The predicted ribosomal binding site (RBS), −10 and −35 promoter elements are highlighted in boldface. Six putative H-NS binding sites (positions −111, −103, +8, +14, +57 and +89) were identified with the Virtual Footprint bacterial promoter analysis tool [35]. (B) Electrophoretic band shift of the amplified 251 bp *f9* promoter and *Taq*I-*Ssp*I digested pBR322 DNA in the presence of various concentrations H-NS (0 μM, 1 μM, 2 μM, 3 μM, 4 μM and 10 μM). Similar to the *bla* promoter positive control, the signal of the *f9* promoter diminishes as its gel migration is impeded by increasing H-NS concentrations, demonstrating that H-NS binds directly to the *f9* promoter sequence. Migration of *bla*-negative pBR322 fragments was not affected by H-NS.

In order to determine whether H-NS influences *f9* gene transcription by directly binding to the promoter region, the *f9* promoter was characterised using 5′-RACE and investigated for H-NS interaction by electrophoretic mobility shift assays. The *f9* transcription start site was identified as a guanine residue, 251 nucleotides upstream of the *f9* major subunit gene start codon (Figure 4A). The transcription start site was preceded by a strong −10 promoter consensus sequence (CATAAT) and a moderate −35 promoter consensus sequence (TAGTCG) with an 18 bp spacer region. *In silico* analysis of the promoter region discerned a ribosomal binding site (RBS) directly upstream of the translation initiation site, and identified six putative H-NS binding motifs at positions −111, −103, +8, +14, +57 and +89 (Figure 4A) [35]. To investigate *f9* promoter/H-NS interactions, the 251 bp promoter region was amplified by PCR and mixed with *Taq*I-*Ssp*I-digested pBR322 DNA (containing the H-NS recognised *bla* promoter). The DNA mixture was incubated with increasing concentrations of purified H-NS protein and analysed by mobility shift electrophoresis. The *f9* promoter region and the positive control *bla*-promoter fragment were equally impeded in gel migration in the presence of increasing concentrations of H-NS (Figure 4B). In contrast, the mobility of pBR322 fragments lacking the *bla*-promoter sequence was not altered in the presence of H-NS. These results demonstrate that H-NS binds to the *f9* promoter region.

### Expression of F9 fimbriae in UPEC CFT073 is temperature-dependent

**Figure 5:**
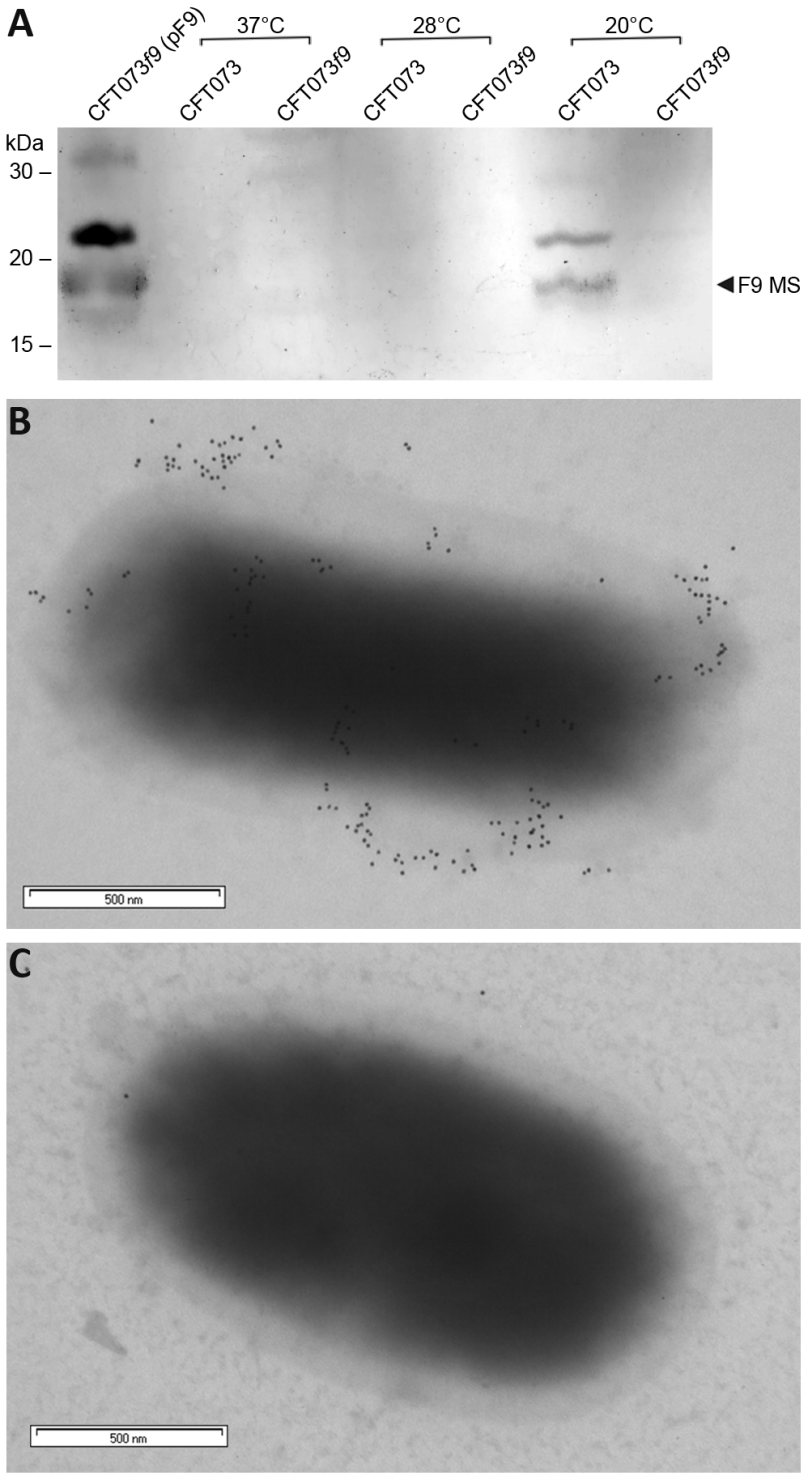
Expression of F9 fimbriae is temperature-dependent. (A) Western blot analysis of wild-type CFT073 and isogenic *f9* null-mutants cultured at various temperatures. The F9 specific antiserum reacts with the F9 mature major subunit protein (∼18.3 kDa) in over-expressing strain CFT073*f9* (pF9). No expression is observed in wild-type CFT073 when cultured at 37°C or 28°C. F9 expression is observed in CFT073 at 20°C, and lost again in isogenic null-mutant CFT073*f9*, illustrating the temperature dependent regulation of F9 fimbriae in UPEC. The mature F9 major subunit (MS) is indicated by an arrow. The 22 kDa higher molecular weight cross-reacting band detected from CFT073 following growth at 20°C is consistent with the size of the unprocessed F9 major subunit protein. TEM micrograph of CFT073*Δ4* (B) and CFT073*Δ4 f9* (C) labelled with immunogold anti-F9 serum after growth at 20°C. Scale bars (500 nm).

The global regulator H-NS modulates the expression of a large subset of genes in response to external stimuli such as temperature [36], [37]. To evaluate whether temperature had an effect on the expression of F9 fimbriae, CFT073 and the isogenic *f9* null mutant were cultured at various temperatures and examined by western blot analysis employing an F9 specific antiserum. No protein bands were detected when CFT073 was cultured at 37°C or 28°C, but at 20°C an 18.3 kDa band corresponding to the mature F9 major subunit was observed (Figure 5). This band was not detected in the CFT073*f9* null-mutant at all temperatures examined, confirming the identity of the band as the F9 major subunit protein (Figure 5). To strengthen these findings we also examined F9 fimbriae expression on the cell surface by immunogold electron microscopy. We detected F9 fimbriae on the surface of CFT073*Δ4* but not CFT073*Δ4 f9* following culture at 20°C (Fig. 5B and 5C). These data represent the first evidence of F9 fimbrial expression by UPEC, and based on the temperature expression profile suggest a role for F9 fimbriae outside the mammalian host.

### F9 fimbriae mediate biofilm formation in UPEC strain CFT073

**Figure 6:**
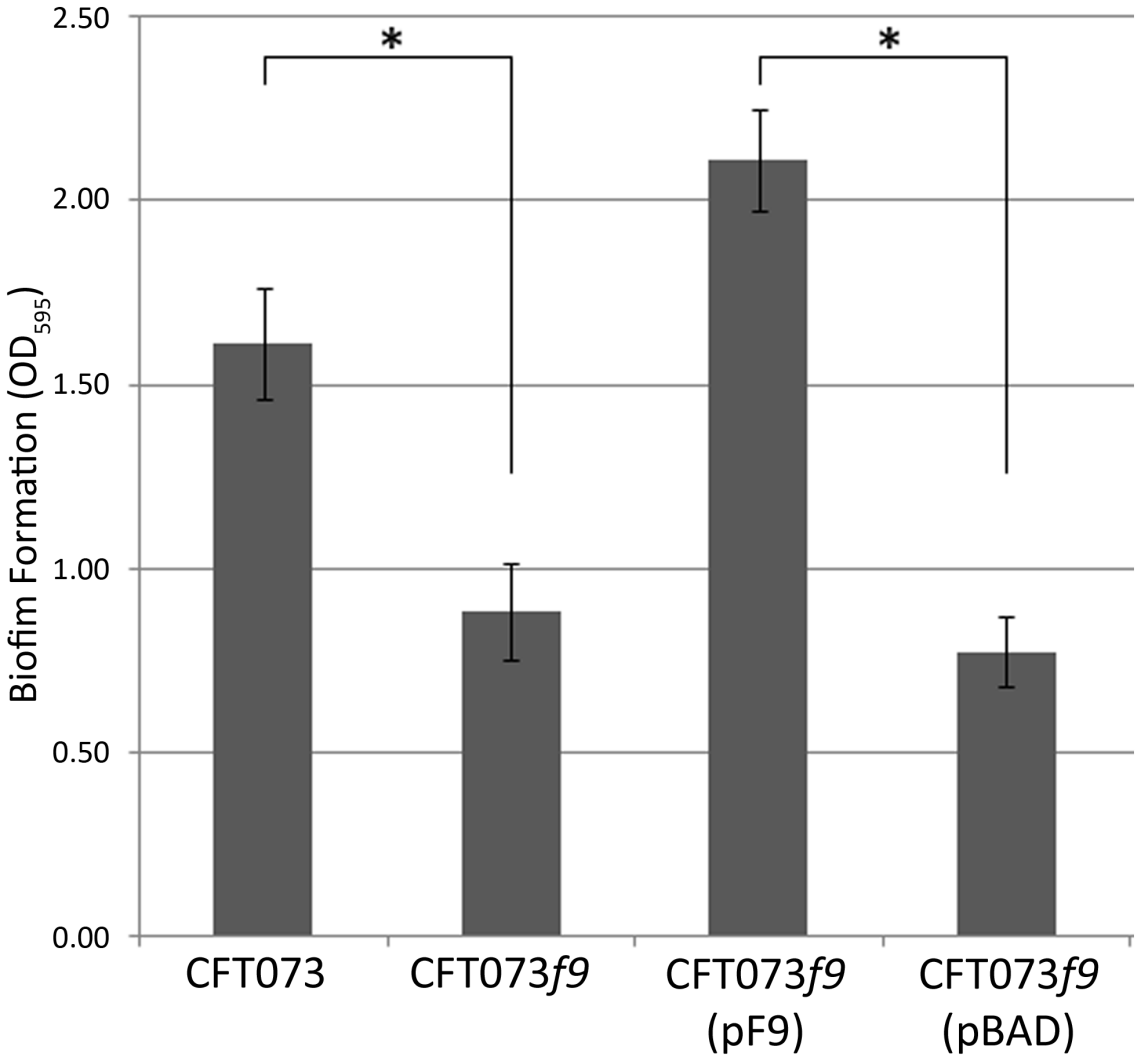
F9 fimbriae mediate biofilm formation in UPEC CFT073 at 20°C. PVC microtitre plate biofilm formation assay of CFT073 and isogenic *f9* null mutants cultured in M9 medium at 20°C. The optical density (OD) at 595 nm (mean ± SD) is an indication of biofilm formation. CFT073 formed a significantly stronger biofilm in comparison to the isogenic *f9* null mutant. The strong biofilm phenotype was restored by complementation of the *f9* deletion mutant with the F9 expression vector pF9 but not with the empty vector pBAD. * Statistically significant (*P*<0.001).

We previously demonstrated that F9 fimbriae mediate a strong biofilm on abiotic surfaces using a plasmid-based system in a recombinant *E. coli* strain [16]. To determine whether F9 fimbriae expressed by wild-type UPEC are involved in biofilm formation, we investigated CFT073 and CFT073*f9* for biofilm formation at 20°C using a microtitre plate assay. Consistent with our F9 fimbrial expression findings using western blot analysis and immunogold-TEM, the expression of F9 fimbriae at 20°C by CFT073 correlated with significant biofilm formation compared to CFT073*f9* under these growth conditions (*P*<0.001; Figure 6). Complementation of CFT073*f9* with the F9 fimbrial expression plasmid pF9 restored the strong biofilm phenotype. These data confirm that F9 fimbriae promote significant biofilm growth on abiotic surfaces by wild-type CFT073 at 20°C.

### The F9 fimbrial adhesin is highly conserved and displays receptor specificity to Galβ1-3GlcNAc terminating glycans

The predicted F9 adhesin is encoded by the last gene in the *f9* operon and contains a characteristic two-domain structure comprising a C-terminal fimbrial integration domain and an N-terminal receptor-binding region. Comparison of the amino acid sequence of the full-length F9 adhesin among the 25 *E. coli* strains that contained an intact *f9* operon revealed a high degree of conservation, with a mean diversity of 0.013±0.004 amino acid substitutions per site over 280 positions. More specific interrogation of the receptor-binding domain of the F9 adhesin revealed even greater amino acid sequence conservation, with a mean diversity of 0.003±0.002 substitutions per site over 160 positions.

The above analysis demonstrates that the F9 adhesin sequence is highly conserved, and indicates that the F9 adhesin from CFT073 can be used to define the overall receptor-binding characteristics of F9 fimbriae. We therefore employed a glycan array in combination with a F9 over-expressing *E. coli* strain labelled with GFP (MS428[pF9, pDW11]) to evaluate the binding specificity of F9 fimbriae to different carbohydrates. In this assay, F9 fimbriae mediated specific binding to Galβ1-3GlcNAc terminating structures, including lacto-*N*-tetraose (Galβ1-3GlcNAcβ1-3Galβ1-4Glc), globotriose (Galα1-4galβ1-4Glc) and the globotriose terminal disaccharide (GalNAcβ1-3Gal) (*P*<0.05). The presence of fucose in Galβ1-3GlcNAc glycans eliminated or reduced affinity by at least 100-fold (data not shown). Of the glycans that were bound by F9 fimbriae, lacto-*N*-tetraose displayed the highest affinity. A glycan competition analysis using 50 μM free lacto-*N*-tetraose resulted in no observable F9 fimbriae-mediated binding to any of the glycans on the array. Taken together, these data provide the first evidence for Galβ1-3GlcNAc glycans as specific receptors for F9 fimbriae, and identify lacto-*N*-tetraose as a high affinity glycan.

## Discussion

Bacterial adhesins mediate attachment to host tissues and abiotic surfaces and provide the first step in colonisation and biofilm formation. Despite the large repertoire of CU fimbriae encoded by UPEC [8], there are only a few well-studied examples of fimbriae that are directly associated with pathogenesis or mediate tissue tropism. Many UPEC fimbriae are cryptic in nature and have not been thoroughly characterised. We previously described F9 fimbriae in UPEC as a functional CU fimbrial type promoting formation of *E. coli* biofilms [16] and have recently demonstrated that they are closely related to the type 1 and F1C/S fimbriae [38], which are both involved in colonisation of the human urinary tract [3]. In this study, the distribution and conservation of F9 fimbriae in diverse *E. coli* lineages was investigated and evaluated in an evolutionary and pathotype associated context. Evolutionary diversity analysis of the F9 adhesin sequence revealed a high conservation of the receptor recognising lectin domain. Furthermore, H-NS was identified as a temperature dependent negative regulator of F9 expression by binding directly to the *f9* promoter region. F9 fimbriae were expressed by CFT073 at 20°C and mediated significant biofilm formation at this temperature. This is the first report of functional F9 expression in wild-type *E. coli*, and provides the first evidence that F9 fimbriae specifically recognise Galβ1-3GlcNAc and lacto-*N*-tetraose glycans.

*E. coli* population genetics have identified five major monophyletic clades (phylogroups A, B1, B2, D and E) [21]. Despite the high frequency of *f9* DNA sequences in the *E. coli* species, the conservation of the *f9* operon between *E. coli* phylogenetic groups varied significantly. A genomic comparison of the *f9* operon from 42 *E. coli* genomes showed that intact *f9* operons were particularly prevalent in phylogenetic group B1 and E, and to a lesser degree in phylogroups B2 and D. In strains from phylogenetic group A, all *f9* operons were disrupted. Variation was also observed among *E. coli* pathotypes, with the *f9* fimbrial genes particularly conserved in intestinal pathogenic isolates representing AIEC, EAEC, EPEC and EHEC, but not ETEC, suggesting a potential role in the pathogenic lifestyle of these bacteria. Indeed, signature-tagged mutagenesis screens using EHEC strains of serotype O157:H7 and O26:H^-^ have previously identified insertion mutants in the *f*9 gene cluster that were significantly impaired for intestinal colonisation in young calves [39], [40]. The *f9* operon was moderately conserved in UPEC genomes. A PCR screen of the 51 isolates in our UPEC collection suggested that the *f9* operon is intact in 80% of the strains, significantly higher than the 61% prevalence of intact *f9* operons in the 72 strains of the diverse and well defined ECOR reference collection. In a phylogenetic context, the results from the *f9* gene prevalence screen of the two collections were consistent with the genomic data. F9 encoding sequences were not found in any other species (except for *Shigella*, a subgenus of *Escherichia*), indicating this fimbrial operon is unique to *E. coli*. The ubiquity of *f9* genes in extant *E. coli* strains suggests that the *f9* operon is ancient and was present in the *E. coli* common ancestor.

H-NS is a histone-like DNA-binding protein that shows affinity for A-T rich and bent nucleation sites on DNA [41]. In this study, several lines of evidence demonstrated a role for H-NS in the regulation of F9 fimbrial expression. In a CFT073 *hns* mutant background, F9 expression was de-repressed, and this effect could be reversed through the introduction of a plasmid containing the *hns* gene. In addition, H-NS bound to a 251 bp DNA fragment containing the mapped *f9* promoter region and a positive control *bla*-promoter fragment with equal affinity. H-NS has been shown to repress multiple other virulence-associated genes in UPEC, including genes encoding alpha-hemolysin, iron uptake systems, fimbriae and autotransporter proteins [33], [41]–[43]. In *E. coli* K-12, several cryptic chaperone-usher fimbrial genes are also repressed by H-NS [34]. The data presented here is the first direct demonstration that H-NS represses F9 fimbriae, and is consistent with a role for H-NS in the regulation of multiple UPEC virulence factors.

F9 fimbriae expression by UPEC CFT073 also displayed a temperature-dependent profile. At 20°C, we detected expression of the F9 major fimbrial subunit protein by western blot and F9 fimbriae structural organelles on the cell surface by immunogold electron microscopy. This F9 expression profile correlated with a strong biofilm phenotype for CFT073 grown under these conditions. Previous studies in EHEC O157:H7 using a chromosomally integrated *lacZ*-*f9* promoter fusion have also suggested F9 temperature-dependent regulation, with stronger activity of the *f9* promoter observed at 28°C versus 37°C [18]. In addition, it has been shown that the expression of the F9 major subunit is increased at 28°C compared to 37°C, however expression levels were too low to detect F9 fimbriae by immunofluorescence [18]. While F9 fimbrial expression in a recombinant *E. coli* K-12 strain promoted enhanced binding to bovine rectal epithelial cells, there were no significant differences in colonization of the terminal rectum of cattle by a wild-type and F9 mutant strain, suggesting that F9 fimbriae are not responsible for EHEC O157 rectal tropism in cattle [18]. Combined, these data suggest that F9 fimbriae contribute to the *E. coli* lifestyle outside the mammalian host, potentially involving colonisation of epidermal surfaces and persistence in the environment through biofilm formation.

Two other types of *E. coli* adhesins that are expressed strongly at 20°C have also been described, namely Mat (or ECP) fimbriae and curli fibres [44], [45]. Mat fimbriae mediate biofilm formation by neonatal meningitis *E. coli* and UPEC at low temperature [46], [47] The expression of Mat fimbriae has also been observed more generally in *E. coli* following cultivation in DMEM, suggesting that temperature-mediated regulation is linked to specific growth conditions [44]. Curli are also strongly expressed at 20°C and associated with biofilm formation [48], however to the best of our knowledge CFT073 has not been shown to produce curli. In our experiments, although the reduction in biofilm formation at 20°C between wild-type CFT073 and the CFT073*f9* mutant was significant, CFT073*f9* still formed a reasonable biofilm (Figure 6). This suggests that CFT073 produces other biofilm formation mechanisms under these conditions, which may include Mat fimbriae. It remains to be determined whether Mat fimbriae are produced by CFT073 at 20°C under the conditions used in our experiments, whether F9 and Mat fimbriae can be co-expressed at 20°C, and if there are additional layers of regulatory control in *E. coli* strains that have the capacity to express both of these fimbriae.

The sequence of the F9 adhesin lectin domain was shown to be highly conserved in *E. coli* strains from different phylogenetic lineages. In order to examine the receptor binding specificity of F9 fimbriae, a glycan array containing 120 structures was utilized. The glycans on the array represented host cell surface glycoconjugates including terminal galactose, mannose, fucosylated and sialylated structures and glycosaminoglycans [31], [32]. These glycans mimic those found on mucosal surfaces, the extracellular matrix, blood antigens and cells of the immune system. The analysis revealed F9 fimbriae bind to Galβ1-3GlcNAc containing glycans, with lacto-*N*-tetraose identified as a high affinity glycan. Epithelial cells of the human urinary tract and kidney are rich in the globoseries glycolipids [49], whereas lacto-*N*-tetraose is a common oligosaccharide found in human milk [50]. In addition, lacto-*N*-tetraose is the oligosaccharide moiety of the lactotetraosylceramide glycosphingolipid receptor present in human gastric epithelium, which is recognised and bound to by *Helicobacter pylori* [51]. Given that many H-NS repressed genes encode virulence factors associated with human infection, it is possible that F9 fimbriae expression in the human host could also contribute to colonisation. In this respect, we were unable to demonstrate binding of a recombinant *E. coli* strain over-expressing F9 fimbriae to human exfoliated urothelial cells, human T24 bladder epithelial cells, human A498 kidney epithelial cells, human Caco-2 intestinal epithelial cells, or human type A red blood cells (data not shown). Thus, the Galβ1-3GlcNAc glycan-containing target cells bound to by F9 fimbriae in the mammalian host remain to be identified.

In conclusion, we have shown that the *f9* fimbriae genes are common to many different *E. coli* lineages and pathotypes and are regulated by H-NS and temperature. F9 fimbriae bind with high affinity to Galβ1-3GlcNAc glycans, including lacto-*N*-tetraose. Finally, UPEC CFT073 expresses F9 fimbriae at 20°C which correlates with strong biofilm formation on abiotic surfaces. Further characterisation of F9 fimbriae is now required to identify its potential role in the colonisation of specific biotic surfaces.

## Supporting Information

Table S1 Primers used in this study.
(DOCX)

Table S2 Glycans screened in this study.
(DOCX)
