# Supplementary material for: F9 Fimbriae of Uropathogenic Escherichia coli Are Expressed at Low Temperature and Recognise Galβ1-3GlcNAc-Containing Glycans
Source: PLoS One. 2014 Mar 26;9(3):e93177. doi: 10.1371/journal.pone.0093177 (PMC3966885; doi:10.1371/journal.pone.0093177)
Supplement: Table S1 — Primers used in this study. (DOCX) [file pone.0093177.s001.docx]

**Table S1: Primers used in this study**

| **Number** | **Sequence (5’ to 3’)** | **Description** |
| --- | --- | --- |
| 2589 | TCGTTTCTGTGTTGGCGATGTC | *f9* major subunit screening (CFT073_c1936) |
| 2590 | ATACCGACATTTTGGGCAGAGC |  |
| 2591 | GGCATATCGTATGCATCAGGTG | *f9* usher screening  (CFT073_c1934) |
| 2592 | GCCAACTACCAATGTTGATGCC |  |
| 2593 | TCAGTTGTAATGTCGATGGCGG | *f9* adhesin screening  (CFT073_c1931) |
| 2594 | TAGGTTGCGCTTAAACCCAGAG |  |
| 2319 | GCCGGGGATCCTCTAGATTTAAGAAGGAG | *gfp* amplification of pKEN2, 5’ BamHI site |
| 2320 | CCGCCGTCGACGGAATTCGTAATCATGGTCA | *gfp* amplification of pKEN2, 5’ SalI site |
| 244 | GTCGATTGAGGATTTCGGATATTGATCTTAAGGCAAAGTGGTGTAGGCTGGAGCTGCTTC | *kan* cassette amplification from pKD4 for type 1(CFT073_c5396) homologous recombination |
| 245 | GCTCCTAACGATACCGTGTTATTCGCTGGAATAATCGTACCATATGAATATCCTCCTTAG |  |
| 416 | TTAAGTTGTGGAAGAACAGCTCTGTCCCGCCTGCATCTCTCCTTTCAGAAGTGTAGGCTGGAGCTGCTTC | *kan* cassette amplification from pKD4 for F1C/S (CFT073_c1237-c1245) homologous recombination |
| 417 | CTAGTTGTAGCTAAAAGTCACATTTATCACTGACTGGACAGCACCGGGCGCATATGAATATCCTCCTTAG |  |
| 789 | AGGACGCCCGCCATAAACTG | *kan* cassette amplification from pKD4 for OE-PCR |
| 790 | GGTTTAACGGTTGTGGACAAC |  |
| 791 | CGTGGATCCAGAAAGCTACC | Downstream amplification of *papX* (CFT073_c3582) for OE-PCR homologous recombination of P1 (c3582-c3593) |
| 792 | GAAGCAGCTCCAGCCTACACGGTTCTCACCACATTATTCGCAGCGGAG |  |
| 793 | CTAAGGAGGATATTCATATGAAAAAGCAGGTATGGTTCAGCGCTCACC | Upstream amplification of *papI* (CFT073_c3593) for OE-PCR homologous recombination of P1(c3582-c3593) |
| 794 | ACTGAATACAAAGTTGCCAGAGAGTGG |  |
| 797 | TGGTCTTTACAAAGCGGAGG | Downstream amplification of *papG_2* (CFT073_c5179) for OE-PCR homologous recombination of P2 (c5179-c5189) |
| 798 | GAAGCAGCTCCAGCCTACACGGCTGGGACTCCATTGTTTCAGTTAACG |  |
| 793 | CTAAGGAGGATATTCATATGAAAAAGCAGGTATGGTTCAGCGCTCACC | Upstream amplification of *papI_2* (CFT073_c5189) for OE-PCR homologous recombination of P2(c5179-c5189 |
| 799 | CCCTAAACCCATCAAACTGG |  |
| 195 | GTGACACCAATGACTGACTGGACGGCTCCGGCTGCAACCTTGTTTCCTGTGTGTAGGCTGGAGCTGCTTC | *kan* cassette amplification from pKD4 for F9 (CFT073_c1931-c1936 ) homologous recombination |
| 196 | GCTGAATGATTGTGATACCAATGTTTCCAGTAATGCAGCTGTTGCATTCCCATATGAATATCCTCCTTAG |  |
| 1583 | TCGTGCGCAGGCAAGAGAATGTACACTTGAAACGCTGGAAGAAATGCTGGGTGTAGGCTGGAGCTGCTTC | *kan* cassette amplification from pKD4 for H-NS (CFT073_c1701) homologous recombination |
| 1584 | TTGATTACAGCTGGAGTACGGCCCTGGCCAGTCCAGGTTTTAGTTTCGCCCATATGAATATCCTCCTTAG |  |
| 4235 | GCGAGAAGAACGAACC | F9GS1 5'RACE |
| 4236 | CGGCATTTACCACTTCACCTT | F9GS2 5'RACE |
| 4237 | TGATTCATCACCCTCGGCT | F9GS3 5'RACE |
| 4296 | GACTCGAGTCGACATCGATTTTTTTTTTTTTTTTT | (dT)17-adaptor primer 5'RACE ([1](#_ENREF_1)) |

**1. 2005. Rapid amplification of 5' complementary DNA ends (5' RACE). Nat Methods 2:629-30.**
