# Supplementary material for: F9 Fimbriae of Uropathogenic Escherichia coli Are Expressed at Low Temperature and Recognise Galβ1-3GlcNAc-Containing Glycans
Source: PLoS One. 2014 Mar 26;9(3):e93177. doi: 10.1371/journal.pone.0093177 (PMC3966885; doi:10.1371/journal.pone.0093177)
Supplement: Table S2 — Glycans screened in this study. (DOCX) [file pone.0093177.s002.docx]

**Table S2: Glycans screened in this study.**

| **Index** | **Name** | **Structure** |
| --- | --- | --- |
|  | **Terminal Galactose** |  |
| 1A. | Lacto-*N*-Biose I | Galβ1-3Glc*N*Ac |
| 1B. | N-Acetyllactosamine | Galβ1-4Glc*N*Ac |
| 1C. | β1-4galactosyl-galactose | Galβ1-4Gal |
| 1D. | β1-6galactosyl-*N*-acetylglucosamine | Galβ1-6Glc*N*Ac |
| 1E. | β1-3galactosyl-*N*-acetylgalactosamine | Galβ1-3Gal*N*Ac |
| 1F. | Galb1-3Gal*N*Acβ1-4Galβ1-4Glc |  |
| 1G | Lacto-*N*-tetraose | Galβ1-3Glc*N*Acβ1-3Galβ1-4Glc |
| 1H | Lacto-*N*-neotetraose | Galβ1-4Glc*N*Acβ1-3Galβ1-4Glc |
| 1I. | Lacto-*N*-neohexose | Galβ1-4Glc*N*Acβ1-6(Galβ1-4Glc*N*Acβ1-3)Galβ1-4Glc |
| 1J. | Lacto-*N*-hexose | Galβ1-4Glc*N*Acβ1-6(Galβ1-3Glc*N*Acβ1-3)Galβ1-4Glc |
| 1K. | Globotriose | Galα1-4Galβ1-4Glc |
| 1L. | Tn Antigen | Gal*N*Acα1-*O*-Ser |
| 1M. | Galactosyl-Tn Antigen | Galα1-3Gal*N*Acα1-*O*-Ser |
| 1N. | α1-3 Galactobiose | Galα1-3Gal |
| 1O. | Linear B-2 Trisaccharide | Galα1-3Galβ1-4Glc*N*Ac |
| 1P. | Linear B-6 Trisaccharide | Galα1-3Galβ1-4Glc |
| 2A. | α1-3, β1-4, α1-3 Galactotetraose | Galα1-3Galβ1-4Galα1-3Gal |
| 2B. | β1-6Galactobiose | Galβ1-6Gal |
| 2C. | Terminal disaccharide of globotriose | Gal*N*Acβ1-3Gal |
| 2D. | Receptor for pili of *P. aeruginosa* | Gal*N*Acβ1-4Gal |
| 2E. | P1 Antigen | Galα1-4Galβ1-4Glc*N*Ac |
| 2F. | α-D-*N*-acetylgalactosaminyl-1-3Gal-β1-4Glc | Gal*N*Acα1-3Galβ1-4Glc |
| 2G. | iso-Lacto-*N*-octose | Galβ1-3Glc*N*Acβ1-3Galβ1-4Glc*N*Acβ1-6(Galβ1-3Glc*N*Acβ1-3)Galβ1-4Glc |
| 2H. | *para*-Lacto-*N*-hexose | Galβ1-3Glc*N*Acβ1-3Galβ1-4Glc*N*Acβ1-3Galβ1-4Glc |
|  |  |  |

|  |  |  |
| --- | --- | --- |
|  | **Terminal *N*`Acetyl glucosamine** |  |
| 4A. | *N,N'*-Diacetyl chitobiose | Glc*N*Acβ1-4Glc*N*Ac |
| 4B. | *N,N',N''*-Triacetyl chitotriose | Glc*N*Acβ1-4Glc*N*Acβ1-4Glc*N*Ac |
| 4C. | *N,N',N'',N'''*-Tetraacetyl chitotetraose | Glc*N*Acβ1-4Glc*N*Acβ1-4Glc*N*Acβ1-4Glc*N*Ac |
| 4D. | *N,N',N'',N''',N'''',N'''''*-Hexaacetyl chitohexose | Glc*N*Acβ1-4Glc*N*Acβ1-4Glc*N*Acβ1-4Glc*N*Acβ1-4Glc*N*Acβ1-4Glc*N*Ac |
| 4E. | Bacterial cell wall muramyl discaccharide | Glc*N*Acβ1-4Mur*N*Ac |
|  |  |  |
|  | **Mannose containing structures** |  |
| 5A. | β1-2-*N*-Acetylglucosamine-mannose | Glc*N*Acβ1-2Man |
| 5B. | Bianntennary *N*-linked core pentasaccharide | Glc*N*Acβ1-2Manα1-6(Glc*N*Acβ1-2Manα1-3)Man |
| 5C. | α1-2-Mannobiose | Manα1-2Man |
| 5D. | α1-3-Mannobiose | Manα1-3Man |
| 5E. | α1-4-Mannobiose | Manα1-4Man |
| 5F. | α1-6-Mannobiose | Manα1-6Man |
| 5G. | α1-3, α1-6-Mannobiose | Manα1-6(Manα1-3)Man |
| 5H. | α1-3, α1-3, α1-6-Mannopentaose | Manα1-6(Manα1-3)Manα1-6(Manα1-3)Man |
|  |  |  |
|  | **Fucosylated structures** |  |
| 7A. | Lacto-*N*-fucopentose I | Fucα1-2Galβ1-3Glc*N*Acβ1-3Galβ1-4Glc |
| 7B. | Lacto-*N*-fucopentose II | Galβ1-3(Fucα1-4)Glc*N*Acβ1-3Galβ1-4Glc |
| 7C. | Lacto-*N*-fucopentose III | Galβ1-4(Fucα1-3)Glc*N*Acβ1-3Galβ1-4Glc |
| 7D. | Lacto-*N*-difucohexose I | Fucα1-2Galβ1-3(Fucα1-4)Glc*N*Acβ1-3Galβ1-4Glc |
| 7E. | Lacto-*N*-difucohexose II | Galβ1-3(Fucα1-4)Glc*N*Acβ1-3Galβ1-4(Fucα1-3)Glc |
| 7F. | H-disaccharide | Fucα1-2Gal |
| 7G. | 2'-Fucosyllactose | Fucα1-2Galβ1-4Glc |
| 7H. | 3'-Fucosyllactose | Galβ1-4(Fucα1-3)Glc |
| 7I. | Lewis^x^ | Galβ1-4(Fucα1-3)Glc*N*Ac |
| 7J. | Lewis^a^ | Galβ1-3(Fucα1-4)Glc*N*Ac |
| 7K. | Blood Group A-trisaccharide | Gal*N*Acα1-3(Fucα1-2)Gal |
| 7L. | Lactodifucotetrose | Fucα1-2Galβ1-4(Fucα1-3)Glc |
| 7M. | Blood Group B-Trisaccharide | Galβ1-3(Fucα1-2)Gal |
| 7N. | Lewis^y^ | Fucα1-2Galβ1-4(Fucα1-3)Glc*N*Ac |
| 7O. | Blood Group H Type II Trisaccharide | Fucα1-2Galβ1-3Glc*N*Ac |
| 7P. | Lewisb tetrasaccharide | Fucα1-2Galβ1-3(Fucα1-4)Glc*N*Ac |
| 8A. | Sulpho Lewis^a^ | SO_3_-3Galβ1-3(Fucα1-4)Glc*N*Ac |
| 8B. | Sulpho Lewis^x^ | SO_3_-3Galβ1-4(Fucα1-3)Glc*N*Ac |
| 8C. | Monofucosyl-para-Lacto-*N-*hexose IV | Galβ1-3Glc*N*Acβ1-3Galβ1-4(Fucα1-3)Glc*N*Acβ1-3Galβ1-4Glc |
| 8D. | Monofucosyllacto-*N*-hexose III | Galβ1-4(Fucα1-3)Glc*N*Acβ1-6(Galβ1-3Glc*N*Acβ1-3)Galβ1-4Glc |
| 8E. | Difucosyllacto-*N*-hexose | Galβ1-4(Fucα1-3)Glc*N*Acβ1-6(Fucα1-2Galβ1-3Glc*N*Acβ1-3)Galβ1-4Glc |
| 8F. | Trifucosyllacto-*N*-hexose | Galβ1-4(Fucα1-3)Glc*N*Acβ1-6(Fucα1-2Galβ1-3(Fucα1-4)Glc*N*Acβ1-3)Galβ1-4Glc |
|  |  |  |
|  | **Neu5Ac containing structures** |  |
| 10A. | Sialyl Lewis^a^ | Neu5Acα2-3Galβ1-3(Fucα1-4)Glc*N*Ac |
| 10B. | Sialyl Lewis^x^ | Neu5Acα2-3Galβ1-4(Fucα1-3)Glc*N*Ac |
| 10C. | Sialyllacto-*N*-tetrose a | Neu5Acα2-3Galβ1-3Glc*N*Acβ1-3Galβ1-4Glc |
| 10D. | Monosialyl, monofucosyllacto-*N*-neohexose | Galβ1-4(Fucα1-3)Glc*N*Acβ1-6(Neu5Acα2-6Galβ1-4Glc*N*Acβ1-3)Galβ1-4Glc |
| 10K. | 2,3'-Sialyllactosamine | Neu5Acα2-3Galβ1-4Glc*N*Ac |
| 10L. | 2,6'-Sialyllactosamine | Neu5Acα2-6Galβ1-4Glc*N*Ac |
| 10M. | LS-Tetrasaccharide a |  |
| 10N. | LS-Tetrasaccharide b | Galβ1-3(Neu5Acα2-6)Glc*N*Acβ1-3Galβ1-4Glc |
| 10O. | LS-Tetrasaccharide c | Neu5Acα2-6Galβ1-4Glc*N*Acβ1-3Galβ1-4Glc |
| 10P. | Disialyllacto-*N*-tetraose | Neu5Acα2-3Galβ1-3(Neu5Acα2-6)Glc*N*Acβ1-3Galβ1-4Glc |
| 11A. | 2,3'-Sialyllactose | Neu5Acα2-3Galβ1-4Glc |
| 11B. | 2,6'-Sialyllactose | Neu5Acα2-6Galβ1-4Glc |
| 11C. | Colominic acid | (Neu5Acα2-8Neu5Ac)n (n<50) |
| 11D. | Biantennary 2,6-sialylated-*N*-glycan-Asn | Neu5Acα2-6Galβ1-4Glc*N*Acβ1-2Manα1-6(Neu5Acα2-6Galβ1-4Glc*N*Acβ1-2Manα1-6)Manβ1-4Glc*N*Acβ1-4Glc*N*Ac-Asn |
|  |  |  |
|  |  |  |
|  |  |  |
|  | **Carageenan and Glycoaminoglycans (GAGS** |  |
| 12A. | Neocarratetraose-41, 3-di-*O*-sulphate (Na^+^) | C_24_H_36_O­_25_S_2_Na_2_ (Mixed anomers. Tetrasaccharide of regular κ - carrageenan) |
| 12B. | Neocarratetraose-41-*O*-sulphate (Na^+^) | C_24_H_37_O_22_SNa (Mixed anomers. Derived from C1003 by removal of the non-reducing terminal 4-sulphate) |
| 12C. | Neocarrahexose-24,41, 3, 5-tetra-*O*-sulphate (Na^+^) | C_36_H_52_O_40_S_4_Na_4_ (Mixed anomers. A hybrid sequence comprising carrageenan disaccharides in the order κ-ι-κ, derived from the carrageenan from *Chondrus crispus*) |
| 12D. | Neocarrahexose-41, 3, 5-tri-*O*-sulphate (Na^+^) | C_36_H_53_O_37_S_3_Na_3_ (Mixed anomers. Hexasaccharide of regular κ-carrageenan) |
| 12E. | Neocarraoctose-41, 3, 5, 7-tetra-*O*-sulphate (Na^+^) | C_48_H_70_O_49_S_4_Na_4_ (Mixed anomers. Octasaccharide of regular κ-carrageenan) |
| 12F. | Neocarradecose-41, 3, 5, 7, 9-penta-*O*-sulphate (Na^+^) | C_60_H_87_O_61_S_5_Na_5_ (Mixed anomers. Decasaccharide of regular κ- carrageenan) |
| 12G. | ΔUA-2S → Glc*N*S-6S Na_4_ (I-S) | C_12_H_15_NO_19_S_3_Na_4_ (Predominant disaccharide produced from heparin by heparinase I and II) |
| 12H. | ΔUA → Gluc*N*S-6S Na_3_ (II-S) | C_12_H_16_NO_16_S_2_Na_3_ (Produced from heparinase II digestion of heparin and heparin sulphate) |
| 12I. | ΔUA → 2S-Glc*N*S Na_3_ (III-S) | C_12_H_16_NO_16_S_2_Na_3_ (Produced from heparin by digestion with heparinase I and II) |
| 12J. | ΔUA → 2S-Glc*N*Ac-6S Na_3_ (I-A) | C_14_H_18_NO_17_S_2_Na_3_ (Minor component produced from heparin by heparinase II) |
| 12K. | ΔUA → Glc*N*Ac-6S Na_2_ (II-A) | C_14_H_19_NO_14_SNa_2_ (Product of the action of heparinases II and III on heparin and heparan sulphate) |
| 12L. | ΔUA → 2S-Glc*N*Ac Na_2_ (III-A) | C_14_H_19_NO_14_SNa_2_ (Minor product of the action of heparinase II on heparin) |
| 12M. | ΔUA → Glc*N*Ac Na (IV-A) | C_14_H_20_NO_11_Na (Produced from heparin sulphate by digestion With heparinase III) |
| 12N. | ΔUA → Gal*N*Ac-4S Na_2_ (ΔDi-4S) | C_14_H_19_NO_14_SNa_2_ (Produced from various chondroitin sulphates By the action of chondroitinases ABC, B and AC-1) |
| 12O. | ΔUA → Gal*N*Ac-6S Na2 (ΔDi-6S) | C_14_H_19_NO_14_SNa_2_ (Produced from various chondroitin sulphates By the action of chondroitinases ABC, AC-1 and C) |
| 12P. | ΔUA → Gal*N*Ac-4S,6S Na_3_ (ΔDi-disE) | C_14_H_18_NO_17_S_2_Na_3_ (Produced from various chondroitin sulphates By the action of chondroitinases ABC, B and AC-1) |
|  |  |  |
| 13A. | ΔUA → 2S-Gal*N*Ac-4S Na_2_ (ΔDi-disB) | C_14_H_18_NO_17_S_2_Na_3_ (Produced from various chondroitin sulphates by action of chondroitinase ABC and/or B. Most typically from chondroitin sulphate B (dermatan sulphate)) |
| 13B. | ΔUA → 2S-Gal*N*Ac-6S Na_3_ (ΔDi-disD) | C_14_H_18_NO_17_S_2_Na_3_ (Produced from various chondroitin sulphates by the action of chondroitinase ABC) |
| 13C. | ΔUA → 2S-Gal*N*Ac-4S-6S Na_4_ (ΔDi-tisS) | C_14_H_17_NO_20_S_3_Na_4_ (Produced as a minor component by the action of chondroitinase ABC on various chondroitin sulphates, particularly B) |
| 13D. | ΔUA → 2S-Gal*N*Ac-6S Na_­2_ (ΔDi-UA2S) | C_14_H_19_NO_14_SNa_2_ (Produced as a minor component from various chondroitin sulphates by the action of chondroitinase ABC) |
| 13E. | ΔUA → Glc*N*Ac Na (ΔDi-HA) | C_14_H_20_NO_11_Na (The only unsaturated disaccharide produced from hyaluronic acid by the action of chondroitinase ABC or AC-1) |
| 13F. | Hyaluronan fragments (4mer) | (GlcAβ1-3Glc*N*Acβ1-4)n (n=4) |
| 13G. | Hyaluronan fragments (8mer) | (GlcAβ1-3Glc*N*Acβ1-4)n (n=8) |
| 13H. | Hyaluronan fragments (10mer) | (GlcAβ1-3Glc*N*Acβ1-4)n (n=10) |
| 13I. | Hyaluronan fragments (12mer) | (GlcAβ1-3Glc*N*Acβ1-4)n (n=12) |
| 13J. | Heparin | (GlcA/IdoAα/β1-4Glc*N*Acα1-4)n (n=200) |
| 13K. | Chondroitin sulfate | (GlcA/IdoAβ1-3(±4/6S)Gal*N*Acβ1-4)n (n<250) |
| 13L. | Dermatan sulfate | ((±2S)GlcA/IdoAα/b1-3(±4S)Gal*N*Acβ1-4)n (n<250) |
| 13M. | Chondroitin 6-Sulfate | (GlcA/IdoAβ1-3(±6S)Gal*N*Acβ1-4)n (n<250) |
| 13N | HA - 4 | (GlcAβ1-3Glc*N*Acβ1-4)n (n=4) |
| 130 | HA - 6 | (GlcAβ1-3Glc*N*Acβ1-4)n (n=6) |
| 13P | HA - 8 | (GlcAβ1-3Glc*N*Acβ1-4)n (n=8) |
| 14A | HA 10 | (GlcAβ1-3Glc*N*Acβ1-4)n (n=10) |
| 14B | HA-12 | (GlcAβ1-3Glc*N*Acβ1-4)n (n=12) |
| 14C | HA-14 | (GlcAβ1-3Glc*N*Acβ1-4)n (n=14) |
| 14D | HA-16 | (GlcAβ1-3Glc*N*Acβ1-4)n (n=16) |
| 14E | HA 30000 Da | (GlcAβ1-3Glc*N*Acβ1-4)n |
| 14F | HA 107000 Da | (GlcAβ1-3Glc*N*Acβ1-4)n |
| 14G | HA 190000 Da | (GlcAβ1-3Glc*N*Acβ1-4)n |
| 14H | HA 220000 Da | (GlcAβ1-3Glc*N*Acβ1-4)n |
| 14I | HA 1600000 Da | (GlcAβ1-3Glc*N*Acβ1-4)n |
| 14J | Heparin sulfate | (GlcA/IdoAα/ IdoASα/β1-4Glc*N*Ac/GlcNS/Glc*N*Ac6Sα1-4)n |
| 14K | b1-3Glucan | (Glcβ1-4Glc)n |
